# Expression and Localization Profiles of Tight Junction Proteins in Immune Cells Depend on Their Activation Status

**DOI:** 10.3390/ijms25094861

**Published:** 2024-04-29

**Authors:** Lena Voges, Franziska Weiß, Ana-Teresa Branco, Michael Fromm, Susanne M. Krug

**Affiliations:** 1 Clinical Physiology/Nutritional Medicine, Charité—Universitätsmedizin Berlin, 12203 Berlin, Germany; 2 Institute of Microbiology, Infectious Diseases and Immunology, Charité—Universitätsmedizin Berlin, 12203 Berlin, Germany

**Keywords:** immune cells, tight junction proteins, claudins, TAMP, angulin, LSR

## Abstract

The ability of the immune system to combat pathogens relies on processes like antigen sampling by dendritic cells and macrophages migrating through endo- and epithelia or penetrating them with their dendrites. In addition, other immune cell subtypes also migrate through the epithelium after activation. For paracellular migration, interactions with tight junctions (TJs) are necessary, and previous studies reported TJ protein expression in several immune cells. Our investigation aimed to characterize, in more detail, the expression profiles of TJ proteins in different immune cells in both naïve and activated states. The mRNA expression analysis revealed distinct expression patterns for TJ proteins, with notable changes, mainly increases, upon activation. At the protein level, LSR appeared predominant, being constitutively present in naïve cell membranes, suggesting roles as a crucial interaction partner. Binding experiments suggested the presence of claudins in the membrane only after stimulation, and claudin-8 translocation to the membrane occurred after stimulation. Our findings suggest a dynamic TJ protein expression in immune cells, implicating diverse functions in response to stimulation, like interaction with TJ proteins or regulatory roles. While further analysis is needed to elucidate the precise roles of TJ proteins, our findings indicate important non-canonical functions of TJ proteins in immune response.

## 1. Introduction

The immune system is the first line of defense against environmental pathogens that enter the body through absorption in the intestines or lungs. In tissues that encounter environmental agents, the immune system must differentiate between beneficial bacteria, e.g., the gut microbiome; harmless substances, e.g., pollen; and harmful agents like pathogenic bacteria or viruses and needs to modulate the immunological response accordingly to maintain homeostasis. Specialized cells such as dendritic cells (DCs) and macrophages are able to sample the apical lumen for antigens by migrating through the underlying epithelium or by penetrating it with their dendrites [1]. The antigen sampling in turn can trigger downstream responses leading to a wide-spread immune reaction. Upon activation, granulocytes, neutrophils, eosinophils, and basophils infiltrate the lumen by migrating through the epithelium. This reaction can be exacerbated in autoimmune conditions such as asthma or inflammatory bowel disease [2,3]. In addition, immune cells that are transported via blood vessels from the lymphatic system to the lamina propria of any particular organ need to pass through endothelial tissue [4]. It has been shown that passage occurs preferentially via the paracellular cleft, and here, a preferential pathway at tricellular contacts has been reported for both neutrophils as well as for T cells [5,6].

The paracellular space is tightened and regulated in its barrier properties by the junctional complex, in particular by the tight junction (TJ). Thus, interactions with components of the TJ are necessary to allow paracellular passage.

TJs are complex multi-protein structures of epi- and endothelia and are located in the most apical site of the lateral membrane, connecting membranes of adjacent cells. Through this, they regulate the permeability properties of the paracellular cleft. The TJ proteins that mainly control permeability comprise the family of claudins (Cldn), which, in mammals, comprise 27 members [7]. Besides their general tightening properties, certain claudins mediate the passage of small cations, anions, and water, through this forming selective paracellular channels (for review see [8,9]). Further TJ protein families are the junctional adhesion molecules (JAMs) and the family of TJ-associated MARVEL proteins (TAMPs), to which occludin, marvelD3 (MD3), and tricellulin belong. Tricellulin has a special role as it is predominantly localized at tricellular TJs (tTJ) that are formed at the tricellular contacts. The central tube of the tricellular TJ (tTJ) with its diameter of 10 nm is assumed to form a weak point of the whole paracellular barrier [10]. Tricellulin, together with the members of the TJ protein family of angulins comprising lipolysis-stimulated lipoprotein receptor (LSR), immunoglobulin-like domain containing receptor (ILDR) 1, and ILDR2 (also called angulin-1, angulin-2, and angulin-3), tightens and regulates the tTJ barrier properties, especially for macromolecules [11,12] and also water [13,14].

The interaction mechanisms of immune cells with TJs are not well understood yet. However, the expression of TJ proteins on different immune cells has been reported already for a long time. For example, DCs express Cldn1, Cldn7, and zonula occludens protein 2 (ZO-2), a TJ-associated scaffold protein [15]. In the human monocytic cells, THP-1, mRNA for occludin, tricellulin, JAM-A, ZO-1, ZO-2 and Cldn4, Cldn7, Cldn8, and Cldn9 have been detected [16]. Tricellulin has been reported in microglia [17] and occludin in astrocytes [18]. In monocyte-derived macrophages and monocyte-derived dendritic cells, occludin, claudin-1, ZO-3, and JAM-A have been found [19] and JAM-A expression has been confirmed on circulating leukocytes and DCs [16]. Here, it is now known that after rolling in blood vessels and attaching to the intercellular adhesion molecule (ICAM) 1 [20], leukocytes may interact with JAM-A via their lymphocyte function-associated antigen (LFA)-1 receptor as the first junctional contact [21]. Furthermore, T cell diapedesis through tTJ in endothelia is dependent on tricellulin [6], and dendrites of Langerhans cells, specific dendritic cells of the skin, can interact with TJs and seal the paracellular space by recruiting tricellulin [22].

The presence of TJ proteins in or on immune cells supports the assumption that immune cells may interact with TJ proteins of the epi- or endothelium via their own subsets of TJ proteins. In this regard, we hypothesize that immune cell subtypes possess specific expression profiles of TJ proteins for their distinct interactions and that these profiles may change upon activation.

To test these hypotheses, we analyzed the expression of TJ proteins in different immune cell subtypes in naïve and stimulated conditions. We found that, indeed, specific expression profiles could be determined in the different immune cells at the mRNA level, of which only a few TJ proteins were also detectable at the protein level. After stimulation, the expression profiles changed and became more homogenous among the immune cell species. Furthermore, we report that, in particular, LSR/angulin-1 seems to be of particular interest for further studies as it was highly expressed in all immune cells and was also found to be located within the membrane, suggesting a function for interaction.

## 2. Results

### 2.1. Primary Peripheral Blood Immune Cells Possess Distinct mRNA Expression Profiles of TJ Proteins That Change upon Stimulation

**Figure 1:**
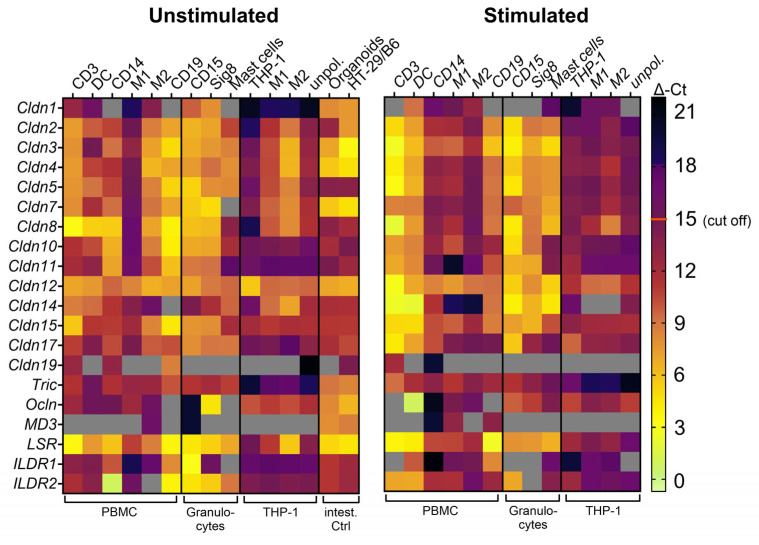
Heat map showing ∆Ct values in different naïve immune cells, THP-1 cell lines, and positive controls in a color-coded manner. Light colors indicate low ∆Ct values (high mRNA expression) and dark colors indicate high ∆Ct values (low mRNA expression). Grey = no detected values. Values higher than ∆Ct = 15 (cut-off) were defined as not-relevant mRNA expression levels. Left: unstimulated cells; right: stimulated cells; LPS: CD3^+^, DC, THP-1; PMA: CD14^+^, mast cells, and THP-1; fMLP: CD15^+^ and Siglec8^+^ (data show results of pooled immune cell samples; *n* = 2–6).

The mRNA expression of several claudins was analyzed in various primary immune cells obtained from peripheral blood, as well as in the immortalized monocyte cell line THP-1. The ∆Ct values relative to the housekeeping gene GAPDH of these analyses were documented in a heat map (Figure 1). These values were assessed for unstimulated and stimulated (with either LPS, PMA, or fMLP) samples. In addition, different positive controls consisting of human colon organoids and the human intestinal epithelial cell line HT-29/B6 were analyzed. In the figure, the lower the ∆Ct value is, the lighter the color is, indicating a higher mRNA expression. In positive controls, no ∆Ct value higher than 15 was detected, and this value was therefore chosen as the cut-off for relevant mRNA expression. In general, mRNA for several TJ proteins was detected in unstimulated primary immune cells where it was at a comparable level to the positive controls, with CD14^+^-derived unstimulated M1 macrophages as an exception.

CD19^+^ cells generally showed the highest levels of TJ protein mRNA for many of the analyzed ones, followed by CD3^+^ cells. Among the claudins, Cldn8 was the one exhibiting highest mRNA expression (∆Ct ≤ 2), and Cldn12 mRNA was detected in all samples.

Regarding the tTJ proteins, LSR (angulin-1) was the only one that showed consistently high expression within most primary immune cells (∆Ct ≤ 5). However, in some cells such as granulocytes and CD14^+^ monocytes, ILDR2 mRNA also was very well detectable (∆Ct ≤ 5). Of further note was that THP-1 cells had no consistent mRNA levels of the studied TJ proteins at all except for Cldn12. In addition, M1 and M2 cells derived from THP-1 also had no comparable mRNA expression profiles to M1 and M2 derived from CD14^+^ monocytes.

When cells were stimulated, the mRNA expression of claudins increased in most immune cells. This was especially noticeable in T cells (CD3^+^) and DCs. However, no specific TJ protein’s mRNA expression appeared to be the mainly regulated one in response to stimulation. In addition, a few TJ proteins among those studied, namely MD3 and Cldn19, were not detected at all or were detected only at very low mRNA levels. Interestingly, Cldn7 mRNA was not detected in unstimulated mast cells but was well detectable after stimulation.

### 2.2. Expression of Some TJ Proteins Can Be Confirmed also at Protein Level

**Figure 2:**
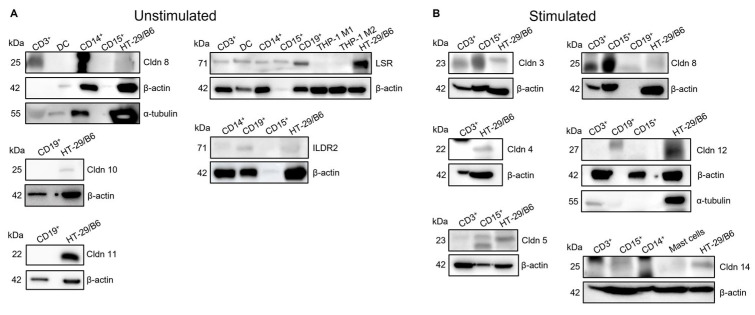
Representative Western blots performed for TJ proteins with highly detected mRNA expression in unstimulated (**A**) and stimulated immune cells (**B**). As endogenous loading controls, β-actin or α-tubulin were used, and as positive controls for TJ protein expression, HT-29/B6 cells were used. Stimulation—LPS: CD3^+^, DC, and THP-1; PMA: CD14^+^, mast cells, and THP-1; fMLP: CD15^+^ and Siglec8^+^ (data show results of pooled immune cell samples; *n* = 2–4).

TJ proteins that showed high abundance at the mRNA level were chosen to be analyzed for protein expression, employing Western blot analysis (Figure 2). However, not all of the potential candidates turned out to be detectable at the protein level, indicating that the presence of mRNA did not necessarily mean translation into proteins. Cldn12, although its mRNA was found in all immune cells, could not be found at the protein level. However, besides the signal for the positive control, a slightly larger band was detected in stimulated CD19^+^ cells that could be a translationally modified Cldn12. The mRNA findings for Cldn8 could be confirmed at the protein level for CD3^+^ cells (unstimulated and stimulated) and, after stimulation, also in CD15^+^ cells.

LSR expression could be confirmed in all tested immune cells except THP-1 and the derived M1 and M2 cells. ILDR2 was expressed in the granulocytes, Siglec8^+^, and CD15^+^, as well as in CD14^+^ monocytes. Interestingly, protein expression levels of ILDR2 were lower, as expected from the mRNA data, in CD14^+^ and CD15^+^ cells, underlining that mRNA expression does not have to correlate well with protein expression.

Regarding the stimulated cells (Figure 2B), Cldn3 was expressed in both CD3^+^ and CD15^+^ cells. Lower molecular weights than expected were detected for Cldn8 in CD3^+^ and CD15^+^ after stimulation. Whether these were modified protein signals or unspecific was not further analyzed. Similar observations were made for Cldn14, which, in general, was less well detectable.

### 2.3. Claudin-Binding Domain of Clostridium Perfringens Enterotoxin May Bind Only to Claudins of Stimulated Immune Cells

**Figure 3:**
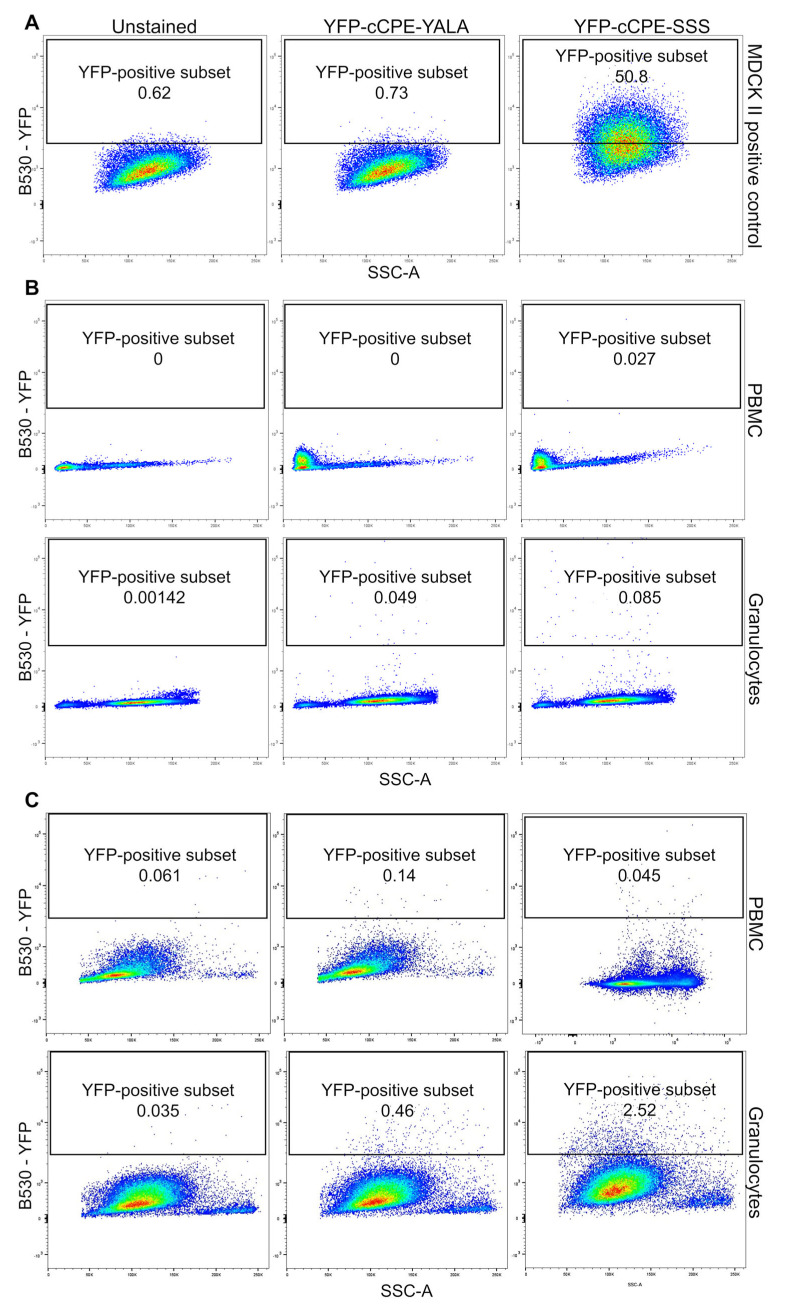
Single cells (either MDCK II, PBMC, or granulocytes) were analyzed for claudin surface expression using YFP-tagged cCPE-SSS and compared against unstained and YFP-tagged cCPE-YALA negative controls. Due to higher mean fluorescence intensity, positively stained cells appeared higher on the y-axis (**A**, right panel) compared to unstained or negative controls (**A**, left and middle panels). Both unstimulated (**B**) as well as stimulated (**C**) PBMCs and granulocytes did not show this upwards shift, suggesting that there is no detectable claudin surface expression in these cells. The framed areas indicate the range in which positively coupled cells are to be expected. The numbers in the frame show the percentage of cells from the initial population detected. On the y-axis, the fluorescence intensity of YFP is plotted against the side scatter on the x-axis (representative images for *n* = 2–3).

As the potential expression of claudins on immune cells might be too low for detection in Western blots, we used the ability of the claudin-binding domain of *Clostridium perfringens* enterotoxin (cCPE) to bind to certain claudins when present on the cell surface. For this, we used a modified variant of cCPE, S305P/S307R/S313H (SSS) [23], which is known to bind to a variety of claudins including Cldn1 to -9, Cldn14, and Cldn19 [23,24,25,26]. As negative controls, unstained cells and, to exclude unspecific binding, the inactive Y306A/L315A (YALA) [23] variant of the aforementioned cCPE were used. Both cCPE variants were labeled with a yellow fluorescent protein (YFP) tag for detection in flow cytometry. As positive controls, MDCK II cells possessing high amounts of different claudins were used. Indeed, the cCPE-SSS bound to MDCK II resulted in a signal intensity shift while negative controls showed no such shift (Figure 3A). This could also be observed in HEK-293 cells, which are claudin-free and thus served as additional negative controls (Supplementary Figure S1). Examining mononuclear cells and granulocytes, no interaction with cCPE-SSS was observed (Figure 3B).

To analyze whether, in immune cells, claudins can be located in the immune cell membrane and whether a higher amount of claudins might be necessary to observe more distinct shifts, T cells were transfected with claudin-4 and then were subjected to the same cCPE experiment before and after stimulation. Here, indeed, the transfected cells showed good binding to cCPE-SSS (Supplementary Figure S2), indicating that all necessary processes were present to allow the transporting of claudins to the membrane.

Furthermore, upon stimulation, a fraction of granulocytes appeared to shift into the YFP-positive range when incubated with cCPE-SSS (unstimulated: 0.095 ± 0.052%, n = 3; stimulated: 3.385 ± 0.865%, n = 2; Figure 3C). This suggested that after activation, not only may expression levels be altered but, also, transport into the membrane might occur.

### 2.4. LSR Is Located in Membranes of Unstimulated Immune Cells While Claudin-8 Only Appears in Membranes after Immune Cell Activation

As the cCPE binding studies suggested that proteins might not be well localized in the membranes of naïve immune cells, immunofluorescent staining was performed and analyzed at different resolution qualities, employing conventional laser-scanning confocal microscopy (LSM) in comparison to stimulated emission depletion (STED) microscopy.

**Figure 4:**
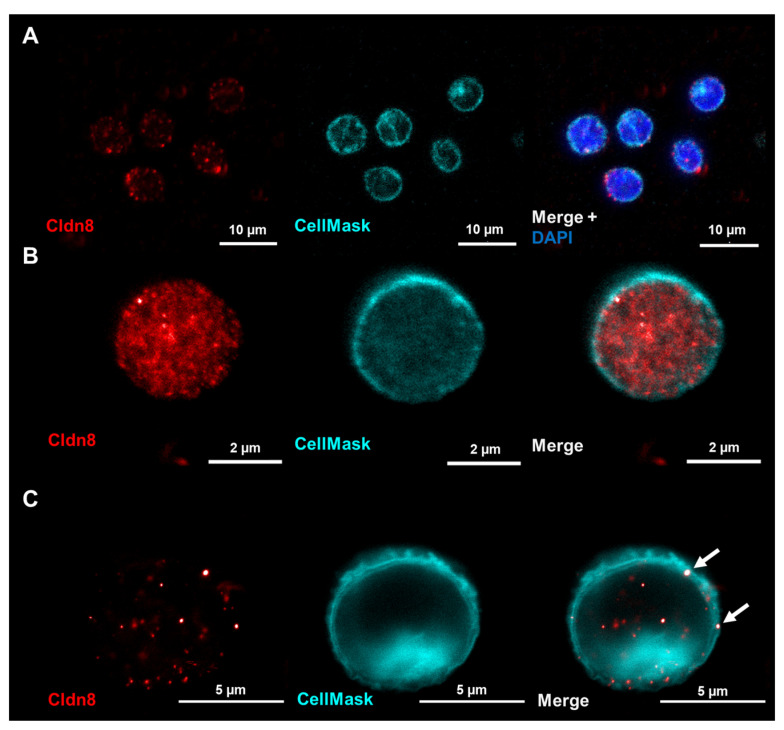
Single CD3^+^ T cells were stained for Cldn8 (red) and the cell membrane was stained using CellMask orange (turquoise). Cells were then analyzed using conventional LSM (**A**) or STED microscopy (**B**,**C**). DAPI (blue) was used to stain nuclei. While unclear in the LSM images, STED analysis revealed Cldn8 to be located in the cytosol of the naïve T cells and only in near vicinity to the membrane. (**C**) In stimulated T cells, the Cldn8 signals appeared in the membrane (arrows) while the diffuse cytosolic signals decreased.

Although the expression of Cldn8 was again confirmed in naïve CD3^+^ T cells through LSM (Figure 4A), the localization appeared to be only in some clusters, maybe attached to the cell membrane. When using the higher resolution offered by STED microscopy, it became more obvious that Cldn8 was only close to the membrane but not located within the membrane surface (Figure 4B), explaining why no binding of cCPE-SSS could be expected. However, when immune cells were activated by stimulation, the localization clearly changed, as in the STED images, distinct Cldn8 signals appeared in the membrane while diffuse signals within the cytosol disappeared, which also would support the findings of the cCPE-SSS experiments (Figure 4C).

**Figure 5:**
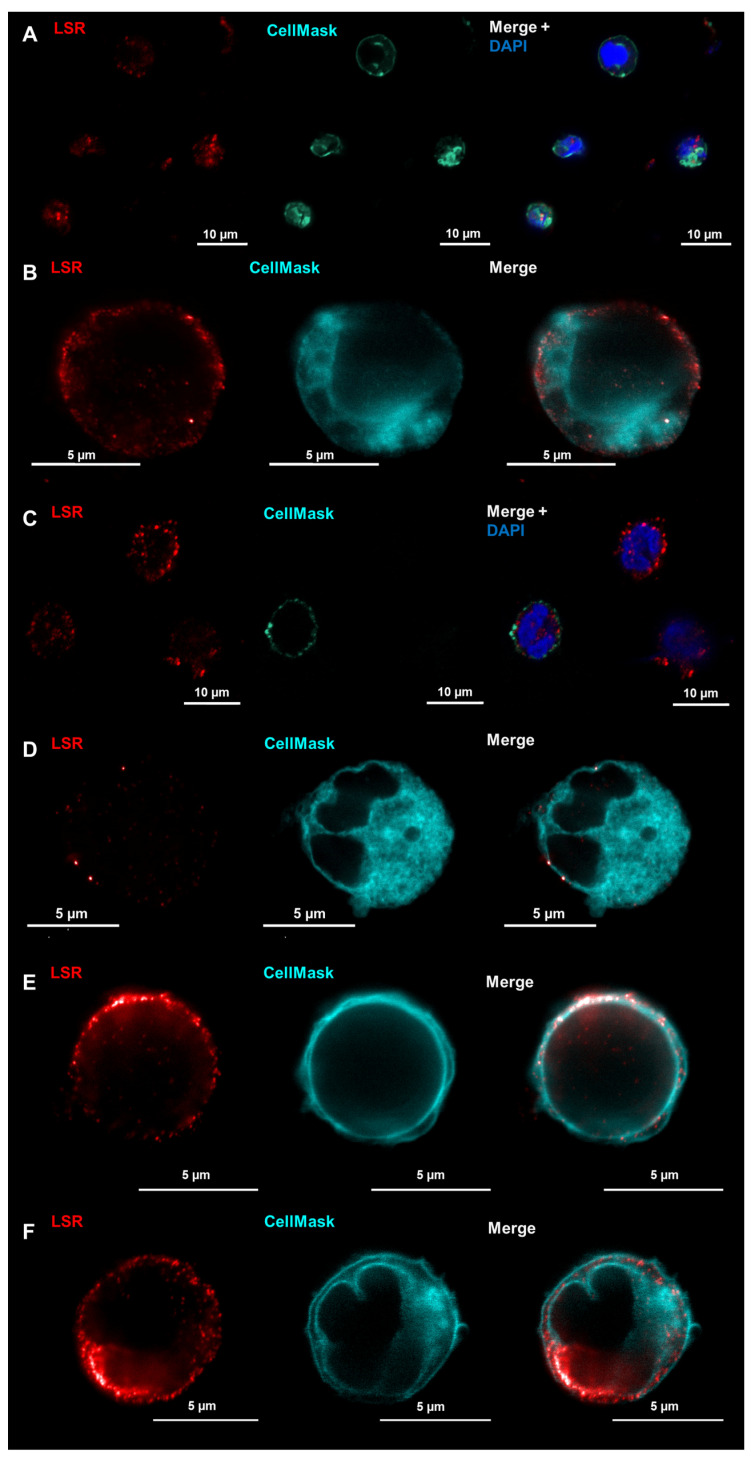
CD3^+^ T cells (**A**,**B**,**E**) and CD15^+^ neutrophils (**C**,**D**,**F**) were stained for LSR (red) and the cell membrane was stained using CellMask orange (turquoise). Cells then were analyzed using LSM (**A**,**C**) or STED microscopy (**B**,**D**–**F**). DAPI (blue) was used to stain nuclei. STED imaging revealed LSR localization both in the cytoplasm as well as in the cell membrane. After stimulation, the signals within the membrane appeared to be even stronger in the membranes of T cells (**E**) and neutrophils (**F**).

The localization of LSR was, like that of Cldn8, vaguely localized at membranes in LSM images of naïve CD3^+^ T cells (Figure 5A) and CD15^+^ neutrophils (Figure 5C). In contrast to that, when using the higher resolution of the STED microscopy, LSR was clearly detectable within the membranes of both cell types besides some cytosolic localization (Figure 5B,D). Upon stimulation, the localization of LSR was still prominent within the cell membranes and appeared even stronger (Figure 5E,F).

## 3. Discussion

The interaction of immune cells with endo- and epithelia is well documented and occurs during antigen uptake, pathogen killing, and migration. However, the actual mode of interaction is not fully understood. Immune cells can pass through the cell layer transcellularly, but a preference for paracellular passage has been observed with an even higher frequency at tricellular contacts [5,6]. This requires direct interaction with the TJ and its proteins. While it has been shown that immune cells can interact with JAM-a via the LFA-1 receptor [21], data for other TJ proteins are still scarce. For example, the involvement of tricellulin in antigen uptake by Langerhans cells has been reported [22].

The necessary immune cell interaction partners remain to be elucidated. Since several studies have reported the expression of TJ proteins in different immune cell subtypes, and, for example, in DCs, Cldn1 and Cldn7 were reported to be upregulated during activation [27], we hypothesized that immune cells may possess specific TJ protein expression patterns. These may allow direct interaction with TJs of endothelia and epithelia, either under physiological conditions or, more importantly, under pathological conditions when the immune system needs to be activated. These expression profiles may also change in the context of activation.

*Immune cells possess specific expression patterns for TJ proteins at the mRNA level that change upon activation, but only a few are expressed at the protein level*

By analyzing the mRNA expression profiles of different immune cell subtypes of PBMCs and granulocytes, we found that each subtype appears to have a specific profile with respect to members of the claudin family, which are also differentially expressed in different tissues. Interestingly, THP-1, which is an immune cell line that can be differentiated into M1- and M2-like immune cells, was not comparable with the freshly isolated immune cells from peripheral blood. This already suggests that such cell lines do not reflect very well the properties of actual immune cells and thus have limited value as a model.

Among the tested claudins, Cldn12 and Cldn8 were detected in nearly all PBMCs and granulocytes, which made them interesting objects for further analysis. Among the two families of TAMPs and angulins, LSR turned out to be detectable at high abundance in all immune cells—to some extent, even in THP-1-derived M1 and M2 cells.

Upon stimulation, the mRNA expression profiles changed. In CD3^+^ T cells, DCs, CD15^+^, mast cells, and Sig8^+^ cells, the expression of claudins was upregulated, suggesting an activation that could also be reflected at the protein level and subsequent interaction possibilities. On the other hand, a general downregulation of claudin mRNA expression was observed in CD14^+^ monocytes and M2 macrophages (in both primary cultures as well as in THP-1-derived cultures). CD14^+^ cells became more similar to M1 macrophages in their expression profile, which could indicate maturation into this cell type.

When testing for the protein expression of the candidates with high mRNA expression, only a few were detectable, which could mean either that expression levels were very low and perhaps irrelevant or that translation was not occurring. Either mRNA degradation is rapid [28], translation could be repressed by regulatory non-coding RNA or miRNA regulation [29], or mRNA is not available because it is localized in other subcellular compartments [30]. In addition, experimental artifacts could lead to mRNA detection. However, one would then not expect to see clear changes that occur in the stimulated samples.

*Claudin-8 is localized within immune cell membranes only upon activation*

At the protein level, we observed that only a few TJ proteins were detectable. Among them, LSR seemed to be the most abundant. However, expression at the protein level did not necessarily imply membrane localization. While LSR was well localized within membranes, Cldn8 was in the cytosol and probably in some vesicles close to the membrane, as indicated by cluster-like signals. Not only high-resolution analysis of immunofluorescence staining with STED but also binding studies with cCPE-SSS suggested that there was no proper localization of claudins in the membranes of unstimulated immune cells. However, upon activation, the localization of at least Cldn8 changed. It appeared inside the membranes, suggesting that it may play a relevant role in the rapid response during immunoregulatory processes, perhaps by interacting with other claudins of the epi- or endothelium. This effect may also be different in different immune cell subtypes. cCPE-SSS binding was detectable to some extent in granulocytes after stimulation, but not in PBMCs.

That claudin localization within the immune cell membranes was activation-dependent suggests roles during the active processes of the immune cells. Upon activation, enhanced interaction with TJs might be necessary to allow increased motility and defense against pathogens. However, this type of interaction may not be limited to the region of TJ localization, as some TJ proteins are known to have extra-junctional localization; in particular, claudin-1, -2, -4, -5, and -7 are often observed to localize in the basolateral membrane below the actual TJ region in epithelia [31,32,33]. Proteins that appear to be upregulated in immune cells upon stimulation are also candidates that are known to be localized extra-junctionally in epithelia, like claudin-1 and -7. These two claudins were already reported to be upregulated in DCs when interacting with the epithelium [27]. However, it remains to be elucidated whether extra-junctional claudins might have a regulatory function, which could be initiated by the interaction with the counterpart claudins of immune cells.

Alternatively, for claudins not being localized in the membranes of immune cells, other functions could be suggested. If claudins are not expressed in the plasma membrane, it would be possible that they might localize in the nucleus and serve regulatory functions as has been shown for claudin-1, -2, -3, and -4 [32,34,35,36,37].

The observed shifts of activation-dependent localization appeared to be immune cell-type specific as we did see such changes in granulocytes in cCPE-SSS experiments but not in PBMCs, suggesting again that the function may be specific for the roles that the respective immune cells have. As we focused on Cldn8, it remains unclear whether other claudins that were expressed at the protein level produce similar localization shifts upon activation and whether each of them has a specific function.

Furthermore, if different activation states can change TJ expression, differentiation states might change it as well since there are many subtypes, especially of mononuclear cells [38,39]. For example, peripheral blood T cells can be progenitor cells to a variety of differently active cells such as natural killer cells, T regulatory cells, or T helper (T_H_) cells, which in turn again can polarize into T_H_1, T_H_2, or T_H_17 when presented with different stages of inflammation [40]. Examining different expression profiles in these various differentiation states could elucidate further the role of TJs in health and disease. The same holds true for tissue-resident immune cells that differentiate according to their destination, such as conventional DCs that are phenotypically different, depending on the lymphoid tissue in which they reside [41], or monocyte progenitor cells that differentiate to monocytes, macrophages, or mast cells [38]. It would be interesting to analyze these expression changes over the course of differentiation and in the context of different tissues, as TJ protein expression is different here as well.

*LSR may have general functions for immune cells, being present in the membranes in unstimulated and stimulated cells*

Based on the observation that LSR was expressed in all analyzed immune cells and was localized within the membrane, one might postulate more general functions of LSR. These might include as initially hypothesized interactions with TJ proteins of endo- or epithelia, maybe especially with tTJ proteins like other angulins. As LSR and angulins in general possess immune globulin-like domains, an interaction similar to actual immune globulins could be assumed.

As shown earlier, neutrophils prefer tricellular contacts to migrate through the endothelial layer [5]. However, the exact mechanisms or interaction partners in the tTJ that facilitate the opening of the TJ and allow for the immune cells to transmigrate remain unclear as well. In their study on T cell diapedesis, Castro Dias et al. could show that when targeting LSR in primary mouse brain microvascular endothelial cells, T cell transmigration via the tTJ was increased while neither crawling nor transmigration time were affected [5,6]. This suggests that there might still be a regulatory mechanism in place despite the endothelial tTJ being strongly impaired without LSR. However, whether LSR on T cells themselves is involved in this regulation needs to be analyzed in the future.

Langerhans cells in the skin are able to recruit tricellulin when their dendrites penetrate a TJ to sample antigens as they need to keep the paracellular space sealed at that point [22]. The actual recruitment mechanisms beyond this are still not known, but considering proteins that are able to recruit tricellulin makes LSR an interesting candidate as it facilitates tricellulin recruitment to the tTJ [42]. Since we could show the membrane location of LSR, it might have a very similar function in other immune cells besides Langerhans cells.

However, regulatory functions may also be possible as it was shown that LSR expressed on tumor cells had a negative effect on CD8^+^ T cell function and activation, which could only be rescued by blocking LSR with antibodies [43].

## 4. Materials and Methods

### 4.1. Immune Cell Isolation

This study was approved by the local ethics committee (No. EA4/015/3). Immune cells were isolated from peripheral human blood drawn from 15 healthy, consenting, voluntary participants into 9 mL blood collection tubes containing EDTA (Greiner Bio-One, Kremsmünster, Austria), adapting a previous published protocol [44], as follows. A quantity of 1.8 mL of 6% (*w/v*) Dextran 70 kDa and 0.9% (*w/v*) NaCl solution was added. After gently inverting the tubes, erythrocytes settled for 1 h and the blood plasma was collected. The plasma was underlayered 1:1 with Ficoll Paque^®^ (Cytiva, Marlborough, MA, USA). Through centrifugation for 20 min at 300× *g* and 4 °C with brakes turned off, the white peripheral blood mononuclear cells (PBMC) were collected and remaining serum and Ficoll Paque^®^ were decanted. The pellet, consisting of granulocytes, was saved and left-over erythrocytes were lysed using 0.2% followed by 1.6% (*w/v*) NaCl. PBMCs and granulocytes were washed and reconstituted in PBS without Mg^2+^ and Ca^2+^ (PBS^w/o^) (Gibco, Thermo Fisher, Waltham, MA, USA).

### 4.2. Cell Culture

#### 4.2.1. T Cells

T cells were cultured at 37 °C and 5% CO_2_ with 20% human serum, 1% insulin–transferrin–selenium (ITS) (Thermo Fisher, Waltham, MA, USA), 100 U/mL penicillin, and 100 µg/mL streptomycin (Corning, Corning, NY, USA) in RPMI medium 1640 with GlutaMAX (Gibco, Thermo Fisher, Waltham, MA, USA). Medium was changed twice a week. Proliferation was induced by adding 100 U/mL interleukin-2 to the medium. Cells were then split after 3 days.

#### 4.2.2. THP-1

THP-1 were cultured at 37 °C and 5% CO_2_ in RPMI medium 1640 with GlutaMAX and 10% FBS, 100 U/mL penicillin, and 100 µg/mL streptomycin added. Differentiation medium contained either 20% human serum, 10% human serum (obtained from donor blood) with 10 ng/mL GM-CSF, or 10% human serum with 10 ng/mL M-CSF in RPMI medium 1640 with GlutaMAX with 100 U/mL penicillin and 100 µg/mL streptomycin added. Medium was changed twice a week.

#### 4.2.3. Monocytes

Monocytes were cultured at 37 °C and 5% CO_2_ with 20% human serum, 100 U/mL penicillin, and 100 µg/mL streptomycin in RPMI medium 1640 with GlutaMAX. Medium was changed twice a week. To polarize monocytes to M1 or M2, macrophages cells were cultured in RPMI medium with 10% human serum, 100 U/mL penicillin, and 100 µg/mL streptomycin and either 10 ng/mL M-CSF or GM-CSF.

#### 4.2.4. B Cells

B cells were cultured at 37 °C and 5% CO_2_ in R5 medium (RPMI 1640 containing 5% human serum, 55 µM 2-mercaptoethanol, 1% L-glutamine, 100 U/mL penicillin, 100 µg/mL streptomycin, 1% HEPES, 1mM sodium pyruvate, and 1% MEM non-essential amino acids). Medium was changed twice a week.

#### 4.2.5. Dendritic Cells

Dendritic cells were cultured in RPMI 1640 medium supplemented with 10% human blood serum, 10 ng/mL GM-CSF, 100 U/mL IL-4, 100 U/mL penicillin, and 100 µg/mL streptomycin at 37 °C and 5% CO_2_. Medium was changed twice a week.

#### 4.2.6. Immune Cell Activation

Immune cells were activated using either 100 ng/mL lipopolysaccharide (LPS) (CD3^+^, DC, THP-1; Merck, Darmstadt, Germany), 20 ng/mL phorbol myristate acetate (PMA) (CD14^+^, mast cells), or 200 ng/mL N-formylmethionyl-leucyl-phenylalanine (fMLP) (CD15^+^, Siglec8^+^) (Sigma-Aldrich, Merck, Darmstadt, Germany) either overnight (CD3^+^, THP-1) or for 3 h at 37 °C and 5% CO_2_.

### 4.3. Fluorescence-Activated Cell Sorting (FACS)

Isolated immune cells were transferred to a 96-well plate and washed with 150 µL PBS^w/o^. After centrifugation for 7 min at 400× *g* and 4 °C, pellets were stained for 10 min on ice for viability using Zombie Aqua™ (BioLegend, San Diego, CA, USA) in PBS^w/o^. After another centrifugation step, cells were stained with the respective antibodies (Table 1) in 2% (*v/v*) FBS (Gibco, Thermo Fisher, Waltham, MA, USA) in PBS^w/o^ at 4 °C until use but for at least 15 min.

*Clostridium perfringens* enterotoxin (cCPE) S305P/S307R/S313H (SSS) and Y306A/L315A (YALA) binding experiments were performed as described before [45]. In brief, cells were incubated with one variant for at least 30 min with a concentration >10 ng/µL.

For the immune-cell positive control, CD3^+^ T cells were nucleofected with human claudin-4. Cultivated T cells were resuspended in 100 mL of the two component nucleofection solution provided in the Lonza Human T cell Nucleofector^®^ kit (Lonza Group, Basel, Switzerland). Adding 5 µg of the expression vector pCMV-4 containing claudin-4 T cells were transferred to the Amaxa Nucleofector II (Lonza Group, Basel, Switzerland) and the program U-024 was used for nucleofection. Cells were immediately transferred to prewarmed RPMI 1640 medium supplemented with 10% FBS. After an incubation period of 24 h, nucleofected T cells were treated with antibodies and cCPE-SSS or cCPE-YALA as described above.

The cells were then either analyzed using the BD LSRFortessa X-20 or sorted using the BD FACS Aria Fusion (BD, Franklin Lakes, NJ, USA). FACS data were evaluated using FlowJo (FlowJo LLC, Becton Dickinson, Ashland, OR, USA). Gating strategy is presented in Supplementary Figure S3.

### 4.4. RNA Isolation and Real-Time qPCR

Cells were pelleted through centrifugation at 500× *g* for 10 min. Supernatant was discarded and pellets were resuspended in 200 µL TRIzol™ reagent (Thermo Fisher, Waltham, MA, USA) per 1,000,000 cells. After 5 min of incubation at room temperature, 0.2 mL chloroform per milliliter TRIzol™ reagent was added, and samples were shaken vigorously for 15 s, then incubated on ice for 10 min. Of the three distinct phases that became visible after centrifugation for 5 min at 12,000× *g* (at 4 °C), the watery top phase was precipitated overnight in 1:1 isopropanol alcohol with 2 µL GlycoBlue™ (Invitrogen, Thermo Fisher, Waltham, MA, USA). After centrifugation for 10 min at 12,000× *g* (4 °C), the resulting pellet was washed twice with 75% ethanol. The pellet was then shortly dried and resolved in 50 µL RNase-free water. A quantity of 4 µg of RNA was reverse-transcribed into cDNA using the High-Capacity cDNA Reverse Transcription Kit (AppliedBiosystems, Thermo Fisher, Waltham, MA, USA) and 1 µL RNase Out™ (Invitrogen, Thermo Fisher, Waltham, MA, USA). A quantity of 1 µL cDNA was used in a 10 µL RT-qPCR mix containing 5 µL NEB Luna probe master-mix (New England Biolabs, Ipswich, MA, USA), 3 µL H_2_O, and 0.5 µL of each FAM and VIC probe (Table 1). Using AppliedBiosystems’s 7500 Fast Real-Time PCR System (AppliedBiosystems, Thermo Fisher, Waltham, MA, USA), samples were analyzed and ∆Ct values relative to the housekeeping gene GAPDH were assessed [46].

### 4.5. Protein Isolation and Western Blotting

Cells were pelleted through centrifugation at 500× *g* for 10 min. Supernatant was discarded and the pellet was resuspended in total lysis buffer (10 mM Tric-HCl (pH 7.5), 150 mM NaCl, 0.5% (*v/v*) Triton X-100, 0.1% (*w/v*) SDS, and protease inhibitors (Complete, Roche, Mannheim, Germany). After 1 h incubation on ice and centrifugation for 15 min at 15,000× *g* (at 4 °C), proteins were collected from the supernatant. After boiling with Laemmli buffer, samples were separated on a 12.5% SDS polyacrylamide gel and transferred onto a PVDF membrane (PerkinElmer, Rodgau, Germany). To detect proteins of interest, the primary antibodies in Table 1 were used. Membranes were washed and incubated overnight with the respective peroxidase-conjugated secondary antibodies before detection. For this, membranes were washed again and incubated in SuperSignal West Pico PLUS (Thermo Fisher, Waltham, MA, USA) and imaged using a Fusion FX6 device. (Vilber Lourmat, Eberhardzell, Germany). 

### 4.6. Immunofluorescence Staining

Immune cells were seeded onto 12 mm poly-L-lysin (Sigma-Aldrich, Merck, Darmstadt, Germany)-coated cover slips. Samples were washed with PBS (Gibco, Thermo Fisher, Waltham, MA, USA) and fixated with 4% PFA for 15 min. 25 mM Glycin was used to quench the PFA, and permeabilization was achieved with 0.2% Triton X-100. Samples were blocked in a solution containing 1% (*w/v*) BSA and 1% (*v/v*) goat serum in PBS with Mg^2+^ and Ca^2+^ (PBS) for 1 h at room temperature. Incubation with primary antibodies overnight at 4 °C and after-washing incubation with secondary antibodies (Table 1) and DAPI (Roche Diagnostics GmbH, Mannheim, Germany) followed. Imaging was performed using a laser-scanning microscope (LSM) (Carl Zeiss AG, Oberkochen, Germany) with excitation wavelengths of 405 nm, 488 nm, 594 nm, and 633 nm.

For STED microscopy, immune cells were seeded onto STED-compatible cover slips and fixated as previously described. Without permeabilization, samples were blocked and then incubated with primary antibodies at 4 °C overnight, and after washing, they were incubated with STED-compatible secondary antibodies and CellMask orange (Table 1). Imaging was performed using a STED microscope (Abberior instruments, Goettingen, Germany) with excitation wavelengths of 574 nm and 647 nm. Images were analyzed using Abberior Imspector (Abberior GmbH, Goettingen, Germany), Zeiss Zen (blue edition 2.6) (Carl Zeiss AG, Oberkochen, Germany), and ImageJ Version 2.15.1.

## 5. Conclusions

The findings of our study show several new aspects for TJ proteins in immunology. We were not only able to confirm reports of TJ protein expression in immune cells but al-so could show that the different immune cells have specific TJ protein expression profiles at the mRNA level. These react to stimulation and may either serve a regulatory function or allow the quick initiation of translation into proteins that then may have cell-type-specific functions. If expressed at the protein level in immune cells, TJ proteins also have different functions. LSR, which we found to be expressed in all immune cells, was localized within the membrane, suggesting roles for interaction, probably with TJ proteins of epi- and endothelia. Claudins appeared not to be per se localized in the membrane, as indicated by the lack of cCPE binding. However, upon activation, the localization changed towards membrane localization. This supported the assumption that TJ proteins may allow increased interaction, which is necessary in a state of immune activation.

In summary, although the exact functions remain to be elucidated in detail, TJ protein expressions within immune cells appear to have diverse functions that depend on the immune cell subtype and status.

## Figures and Tables

**Table 1:** Antibodies and probes used in this study. Abbreviations—CAT: catalogue number; FACS: fluorescence-activated cell sorting; FAM: fluorescein amidite; hu: human; IF: immunofluorescence staining; VIC: Victoria; 2′-chloro7′phenyl-1,4-dichloro-6-carboxyflourescein; WB: Western blotting.

**Primary antibodies and fluorescence labels**		
**Antibody**	**Concentration**	**Supplier**
Anti-huβ-actin	WB 1:1000	Sigma-Aldrich Chemie GmbH, Traufkirchen, Germany, CAT: A544
Anti-huCD1c PB	FACS 1:200	BioLegend, San Diego, CA, USA, CAT: 331507
Anti-huCD3 FITC	FACS 1:200	BioLegend, San Diego, CA, USA, CAT: 317306
Anti-huCD3 PE	FACS 1:200	BioLegend, San Diego, CA, USA, CAT: 300307
Anti-huCD14 AF700	FACS 1:1600	BioLegend, San Diego, CA, USA, CAT: 367113
Anti-huCD11c PE	FACS 1:100	BioLegend, San Diego, CA, USA, CAT: 301605
Anti-huCD19 PB	FACS 1:50	BioLegend, San Diego, CA, USA, CAT: 363035
Anti-huCD19 PerCP	FACS 1:200	BioLegend, San Diego, CA, USA, CAT: 363013
Anti-huCD15 PE	FACS 1:50	BioLegend, San Diego, CA, USA, CAT: 301905
Anti-huCD123 APC	FACS 1:50	BioLegend, San Diego, CA, USA, CAT: 306011
Anti-huCD303 PE	FACS 1:200	BioLegend, San Diego, CA, USA, CAT: 354203
Anti-huClaudin-**3**	WB 1:1000	Invitrogen, Thermo Fisher Scientific GmbH, Dreireich, Germany, CAT: 325600
Anti-huClaudin-5	WB 1:1000	Invitrogen, Thermo Fisher Scientific GmbH, Dreireich, Germany, CAT: MA5-32614
Anti-huClaudin-8	IF 1:200 WB 1:1000	Invitrogen, Thermo Fisher Scientific GmbH, Dreireich, Germany, CAT: 710222
Anti-huClaudin-11	WB 1:1000	BiCell Scientific, Maryland Heights, MO, USA, CAT: 00211
Anti-huClaudin-12	WB 1:1000	IBL Interantional GmbH, Hamburg, Germany, CAT: JP18801
Anti-huClaudin-14	WB 1:1000	Invitrogen, Thermo Fisher Scientific GmbH, Dreireich, Germany, CAT: 36-4200
Anti-huILDR2	WB 1:1000	Sigma-Aldrich Chemie GmbH, Traufkirchen, Germany, CAT: HPA012815
Anti-huLSR	IF 1:200 WB 1:1000	Atlas antibodies AB, Bromma, Sweden, CAT: HPA007270
Anti-huSiglec8 FITC	WB 1:1600	Miltenyi Biotec, Bergisch Gladbach, Deutschland, CAT: 130-098-716
**Secondary antibodies and fluorescence labels**		
**Antibody**	**Concentration**	**Supplier**
4′,6-diamidino-2-phenylindole (DAPI)	IF 1:500	Roche Diagnostics GmbH, Mannheim, Germany, Cat. No.: 10236276001
Anti-mouse	WB 1:10,000	Invitrogen, Thermo Fisher Scientific GmbH, Dreireich, Germany, CAT: 31430
Anti-rabbit	WB 1:10,000	Invitrogen, Thermo Fisher Scientific GmbH, Dreireich, Germany, CAT: 31460
Anti-rabbit AF647	IF 1:250	Invitrogen, Thermo Fisher Scientific GmbH, Dreireich, Germany, CAT: A-21245
Anti-rabbit STARred	IF 1:250	Abberior GmbH, Göttingen, Germany, CAT: STRED-1002-500UG
CellMask™ orange	IF 1:5000	Thermo Fisher, Waltham, MA, USA, Cat. No.: C37608
YFP-cCPE-SSS	FACS 10 ng/mL	Kind gift of PD Dr. Jörg Piontek, Charité—Universitätsmedizin Berlin, Clinical Physiology/Nutritional Medicine
YFP-cCPE-YALA	FACS 10 ng/mL	Kind gift of PD Dr. Jörg Piontek, Charité—Universitätsmedizin Berlin, Clinical Physiology/Nutritional Medicine
Zombie Aqua™ Fixable Viability Kit	FACS 1:1000	BioLegend, San Diego, CA, USA, Cat. No.: 423101
**RT-qPCR Probes**		
**Probe**	**Dye**	**Assay ID**
huClaudin-1	FAM	Hs00912957_m1
huClaudin-2	FAM	Hs00252666_s1
huClaudin-3	FAM	Hs00265816_s1
huClaudin-4	FAM	Hs00976831_s1
huClaudin-5	FAM	Hs00533949_s1
huClaudin-7	FAM	Hs00600772_s1
huClaudin-8	FAM	Hs00273282_s1
huClaudin-10	FAM	Hs00199599_m1
huClaudin-11	FAM	Hs00912957_m1
huClaudin-12	FAM	Hs00273258_s1
huClaudin-14	FAM	Hs00377953_m1
huClaudin-15	FAM	Hs00204982_m1
huClaudin-17	FAM	Hs00273276_s1
huClaudin-19	FAM	Hs00961709_m1
huGAPDH	VIC	4310884E
huILDR1	FAM	Hs01111437_m1
huILDR2	FAM	Hs01025498_m1
huLSR	FAM	Hs01076319_g1
huOccludin	FAM	Hs01049883_m1
huMarvelD3	FAM	Hs00369354_m1
huTricellulin	FAM	Hs00930631_m1

## Data Availability

The data are presented in this paper and are also available on request from the corresponding author.

## Supplementary Materials

The following supporting information can be downloaded at: https://www.mdpi.com/article/10.3390/ijms25094861/s1.
