# Supplementary material for: Expression and Localization Profiles of Tight Junction Proteins in Immune Cells Depend on Their Activation Status
Source: Int J Mol Sci. 2024 Apr 29;25(9):4861. doi: 10.3390/ijms25094861 (PMC11084252; doi:10.3390/ijms25094861)
Supplement: Supplementary file 1 [file ijms-25-04861-s001.zip › ijms-2964516-supplementary.pdf]

Supplementary Figures

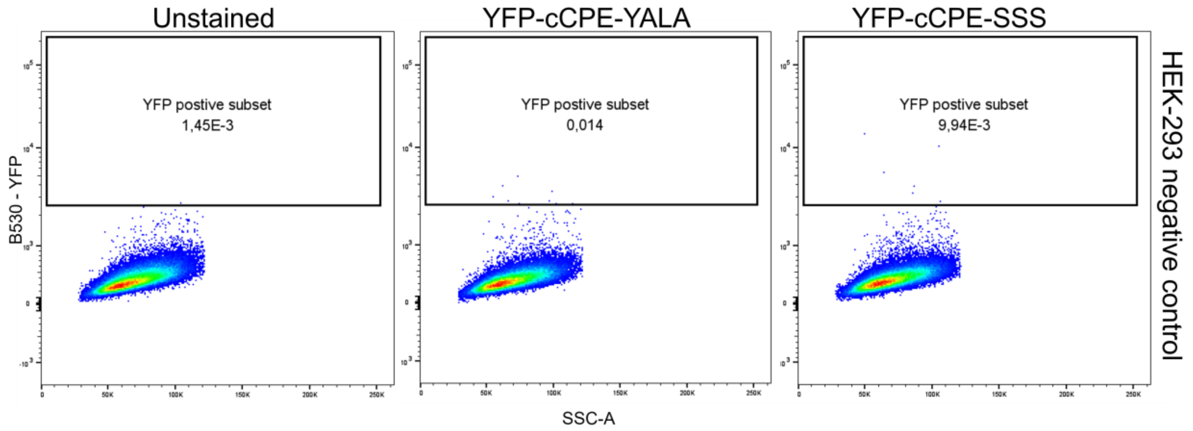

Supplementary Figure S1. HEK-293 cells were analyzed for claudin surface expression using YFP-tagged cCPE-SSS and compared against unstained and YFP-tagged cCPE-Yala negative controls. Due to higher mean fluorescence intensity, positively stained cells will appear higher on the y-axis compared to unstained or negative controls. In this case there was no positive signal because the HEK-293 do not express claudin proteins. On the Y-Axis, the fluorescence intensity of YFP is plotted against the side scatter on the X-axis and numbers in the frame show the percentage of cells from the main population detected in this area.

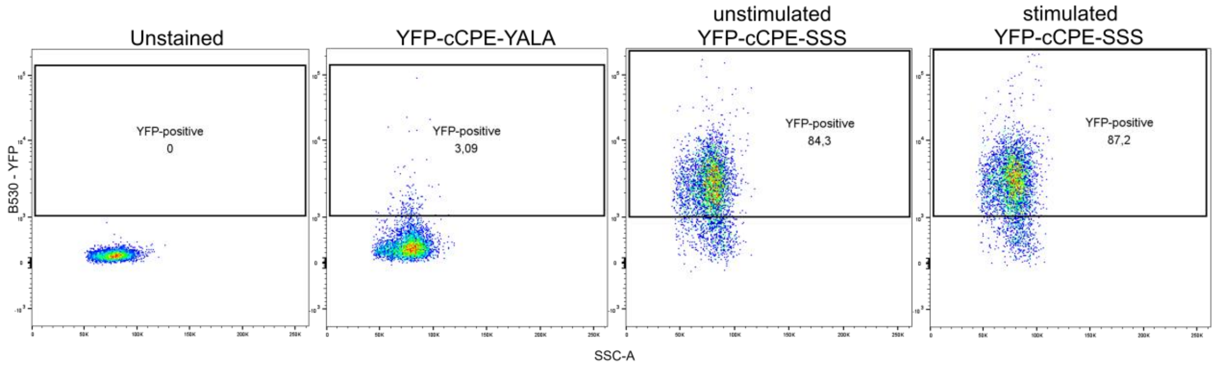

Supplementary Figure S2. Single T cells were transfected with claudin-4 and then analyzed for claudin surface expression using YFP-tagged cCPE-SSS and compared against unstained and YFP-tagged cCPE-YALA negative controls. Unstimulated as well as stimulated T cells bound to cCPE-SSS as indicated by the shift in fluorescence, however. Framed areas indicate the range in which positively coupled cells are to be expected. The numbers in the frame show the percentage of cells from the initial population detected. On the Y-axis, the fluorescence intensity of YFP is plotted against the side scatter on the X-axis.

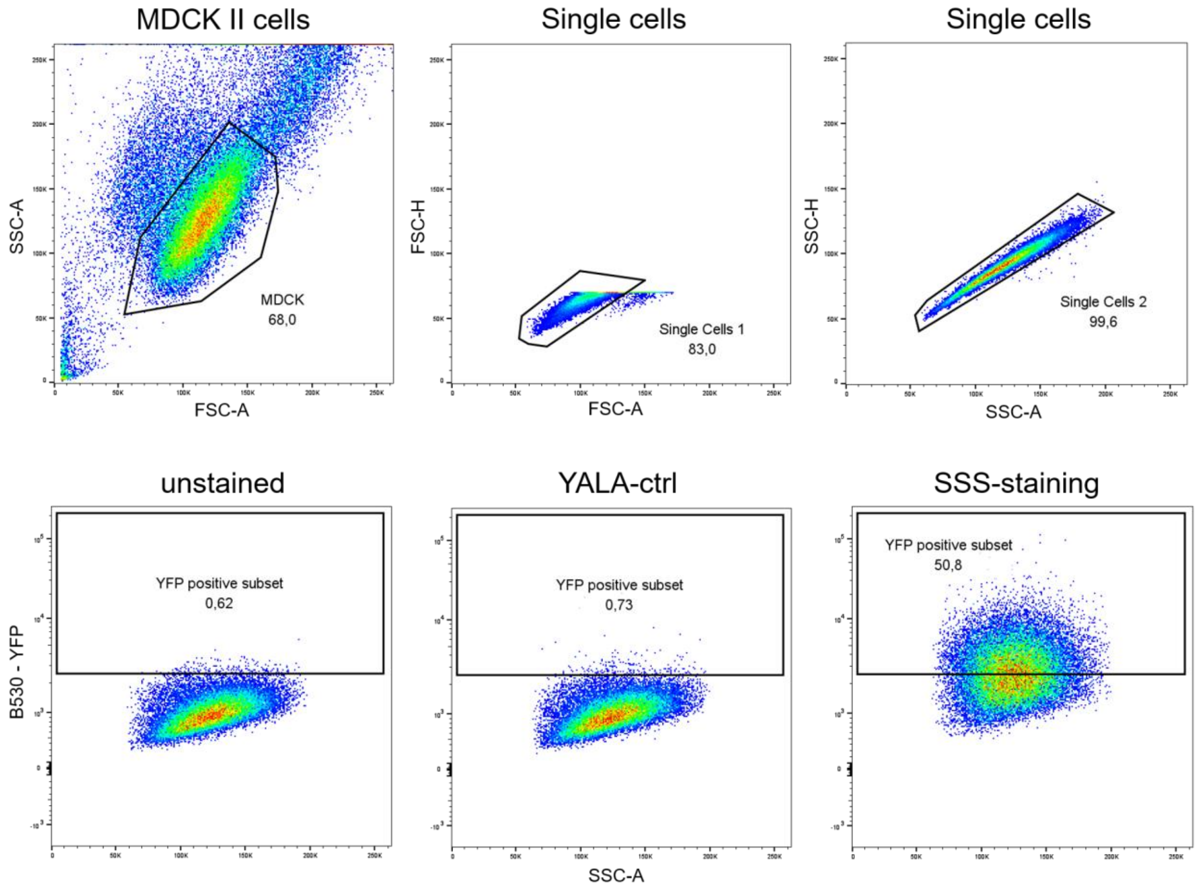

Supplementary Figure S3. Gating strategy shown exemplary for MDCKII cells. First, the whole population was selected. Afterwards any doublets were excluded and at then, any YFP-positive stained cells were identified, signifying cCPE-SSS binding. This gating strategy was used to analyze all the data.
